# Identification of hypoxic-related lncRNAs prognostic model for revealing clinical prognostic and immune infiltration characteristic of cutaneous melanoma

**DOI:** 10.18632/aging.205556

**Published:** 2024-02-15

**Authors:** Congjuan Liao, Jiabao Yang, Liuting Chen, Zhiguang Ye

**Affiliations:** 1 Dermatology and STD Department of The Second Affiliated Hospital, School of Medicine, The Chinese University of Hong Kong, Shenzhen and Longgang District People’s Hospital of Shenzhen, Shenzhen 518172, China; 2 Beijing University of Chinese Medicine Shenzhen Hospital (Long Gang), Shenzhen 518116, China

**Keywords:** cutaneous melanoma, hypoxia, immune infiltration, risk stratification, immunotherapy evaluation

## Abstract

Background: Cutaneous melanoma (CM) remains a significant threat to human health. There are clues to the potential role of hypoxia in CM progression. However, the role of hypoxia-related lncRNAs (HRLs) in CM has not been clarified.

Methods: We obtained hypoxia related genes from MSigDB database and subsequently identified HRLs by applying TCGA database. LASSO-univariate and multivariate Cox analysis were used to comprehensively analyze the survival characteristics and HRLs expressions, and a novel HRLs-related prognostic risk model was subsequently established for comprehensive analysis.

Results: The established risk model could evaluate the clinical outcome of CM accurately. The ability of the model-related risk score was also validated as an independent prognostic indicator of CM. Immune infiltration, TMB analysis, drug sensitivity analysis and immunotherapy evaluation were conducted to comprehensively assess the possible causes of the difference in prognosis. The reliability of bioinformatics results was partially verified by RT-qPCR.

Conclusion: We established a new HRLs related risk model and discussed the potential role of hypoxia in the development of CM, which provided a novel basis for CM risk stratification.

## INTRODUCTION

Cutaneous melanoma (CM) originates from melanocytes in the skin and is the dominate type of melanoma [1]. Due to its high aggressiveness and significant resistance to chemotherapy drugs, CM accounts for 80% of skin cancer deaths [2]. Although immune checkpoint inhibitors have made great progress in the treatment of melanoma [3], the continued increase in CM incidence and mortality drives us to further explore the mechanisms of melanoma development and potential treatments [4].

Hypoxia is one common feature in many solid tumor types, including CM. Compared with cells in a physiological state, hypoxic cancer cells increased glycolysis and reduced oxidative phosphorylation [5]. A pooled dataset of melanoma patients found that the presence of hypoxia within the tumor mass was positively associated with poor outcomes in these patients [5]. Hypoxia affects CM development through multiple mechanisms. As one main regulator of hypoxia response, hypoxia-inducible factor (HIF) consists of two subunits, HIF-α and HIF-β, which are able to bind to hypoxia response elements in DNA sequences [6]. A variety of modifications can regulate the adaptation to anoxic environment by controlling the stability and transcriptional activity of HIF-1α [7]. Through the activation of PI3K, MAPK, NF-kB and other signaling pathways that promote tumor development, HIF-1α responds to growth factor and cytokine stimulation and promotes the survival of cancer cells in hypoxic environments [8]. In addition, when cells trigger HIF-1-dependent pathways under hypoxia conditions, neovascularization occurs [8]. The alteration of HIF-1a expression level in anoxic microenvironment decreased the expression of melanocyte marker and increased its invasiveness in melanoma cells [9].

In addition to HIF-1α, some evidence suggests other possible mechanisms by which hypoxia affects CM development. Hypoxic-induced cancer stem cell-like cells (CSCs) can recruit endothelial progenitor cells to construct pathological vasculature [10]. Metabolic reprogramming induced by low oxygen levels also influenced CM development and was associated with prognosis [11, 12]. In addition, hypoxia induces mutations in the gene encoding p53 through the AKT signaling pathway and is associated with poorer prognosis in melanoma patients [13]. Exposure to hypoxia and high glucose concentrations in mouse CM models showed upregulated expression of galectin-3 and prevented tumor cell apoptosis [14]. Elevated levels of Bcl-2 interacting protein 3 (BNIP3) were detected in hypoxic melanoma cells, which was associated with reduced anti-PD-1 therapeutic response due to induced autophagy [15].

Although above evidences suggest a carcinogenesis role of hypoxia in CM. However, oxidative phosphorylation can be enhanced in some types of CM [16]. At the same time, several drugs that inhibit oxidative phosphorylation may be used to target specific subtypes of melanoma [17]. The presence of higher mitochondrial activity in metastatic cancer than in primary cancer also suggests a potential role for oxidative phosphorylation in tumor metastasis [18, 19]. Therefore, it is still necessary to further probe into the significance of hypoxia in the development of CM.

Hypoxia is a prominent feature of the tumor microenvironment (TME) and is also considered to be an important factor in immune escape [20]. The effects of hypoxia on TME include: impaired T cell infiltration, induced immunosuppression and immune tolerance, induced resistance to cell-mediated cytotoxicity and induced lymphocyte killing activity [21, 22]. Hypoxia has received increasing attention by modulating the role of TME in cancer treatment [23]. In melanoma, targeting HIF-1α has been reported to drive cytotoxic immune effector cells into the tumor and improve combination immunotherapy [20]. In addition, the effect of hypoxia and its metabolites on PD-L1 also suggests its role in CM immunotherapy [24, 25]. Further analysis of hypoxia related TME is of positive significance.

In this study, we subtyped CM patients in the database based on HRLs and subsequently established a corresponding risk model. The effects of the novel risk model on immune infiltration and drug sensitivity were also analyzed to explore the role of hypoxia in the carcinogenesis of CM from a new perspective.

## MATERIALS AND METHODS

### Collection of CM transcriptome matrices

The gene expression files of CM patient samples were acquired from public database The Cancer Genome Atlas Program (TCGA) under the Perl language environment. Utilizing the Perl script, we extracted the gene expression file of each CM samples and merged into a file. CM clinical information was also extracted from the TCGA database using Perl scripts and subsequently merged into the final file. Considering the lack of information on CM sample’s survival, the samples without OS time or less than 0 were deleted. A total of 454 CM samples were screened for subsequent analysis.

### Calculation and identification of HRLs

The hypoxia genes were acquired from the MSigDB database (Supplementary Table 1) [26–28]. The coefficient of hypoxia genes and lncRNAs was calculated by using Pearson correlation algorithm. The selection threshold for identifying the HRLs was setting at |r| >0.5, *p* < 0.001.

### Prognosis characteristic and consensus clustering subtype exploration

Integrated analysis of survival characteristics and HRLs expression in CM samples, the LASSO-univariate Cox algorithm was conducted to evaluate the prognostic value of HRLs. Moreover, the multivariate Cox analysis of HRLs was used to calculate the CM independent prognostic feature. For CM samples clustering, “ConsensusClusterPlus” script was performed based on the independent prognostic feature according to the optimal classification.

### Establishment of HRLs score for CM

Based on the coefficient and expression feature of independent HRLs variables, the HRLs scores were established for CM samples. The optimal survival cutoff helped the CM samples to be classified into the low- and high HRLs score subtypes. To validate the independence of HRLs score for CM prognosis predicting, the training and testing subtypes were divided according to a 7:3 classification threshold by the “caret” package application [29, 30].

### Immune microenvironment characteristic estimation

Based on the transcriptome matrices of CM samples, we estimated the immune microenvironment characteristic of CM samples utilizing the ssGSEA algorithm based on “GSVA” script. “ESTIMATE” script was employed to estimate the immune status of CM samples. The KEGG terms were calculated by the “GSVA” algorithm based on the “c2.cp.kegg.v7.2.symbols.gmt” file.

### TMB landscape and immunotherapy evaluation

The tumor mutation burden (TMB) files of CM samples were downloaded and extracted from the public TCGA databased using the Perl script. Using the “maftools” script to exhibit the landscape of TMB for CM samples. The IPS file of CM was obtained from The Cancer Immunome Database (TCIA). The Tumor Immune Dysfunction and Exclusion (TIDE) database was used for TIDE score evaluating (http://tide.dfci.harvard.edu/).

### RT-qPCR analysis

In this study, we employed RT-qPCR to assess the expression levels of the screened prognostic HRLs. RNA was isolated by TRIZOL (Thermo Fisher Scientific, USA) and Bestar™ qPCR RT Kit (DBI Bioscience, China) was used for cDNA synthesis following the manufacturer’s instructions from HFB4 and A375 cell lines. The relative gene expressions were calculated by the ΔΔCt method, normalizing to the expression of the reference gene and a control sample (Supplementary Table 2).

### Statistical data analysis

All program performance and data analysis were performed in R language environment. For two groups comparison, *T* test and Wilcoxon rank-sum test were used for statistics. One-way ANOVA analysis was applied for statistical analysis among multiple groups. Pearson correlation analysis was used to calculate the correlation between two components and *p* less than 0.05 was regarded as statistically significant.

## RESULTS

### The HRLs signature construction

**Figure 1:**
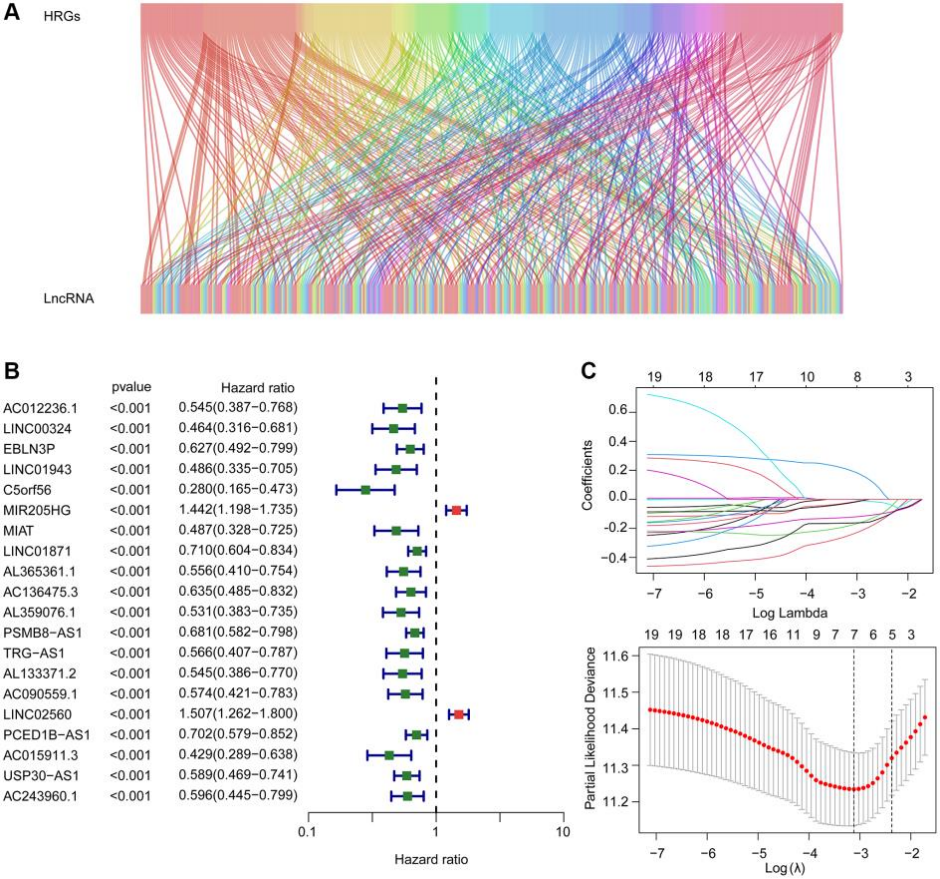
**Risk model construction based on the prognostic HRLs in CM.** (**A**) The Sankey diagram shows the detailed connection between HRGs and HRLs. (**B**) Univariate Cox regression analysis of HRLs. (**C**) LASSO regression analysis displays the minimum lambda and optimal coefficients of prognostic HRLs.

The Sankey diagram visualized the relationship between hypoxia related genes and HRLs (Figure 1A). By the least absolute shrinkage and selection operator (LASSO) utilizing, seven prognostic HRLs associated with the overall survival (OS) rate were identified based on the univariate Cox regression (Figure 1B, 1C). The multivariate Cox regression analyses subsequently selected four prognostic HRLs which differentially expressed in tumor and could independently evaluate the prognosis of CM.

### Consensus clustering analysis and immune infiltration landscape

**Figure 2:**
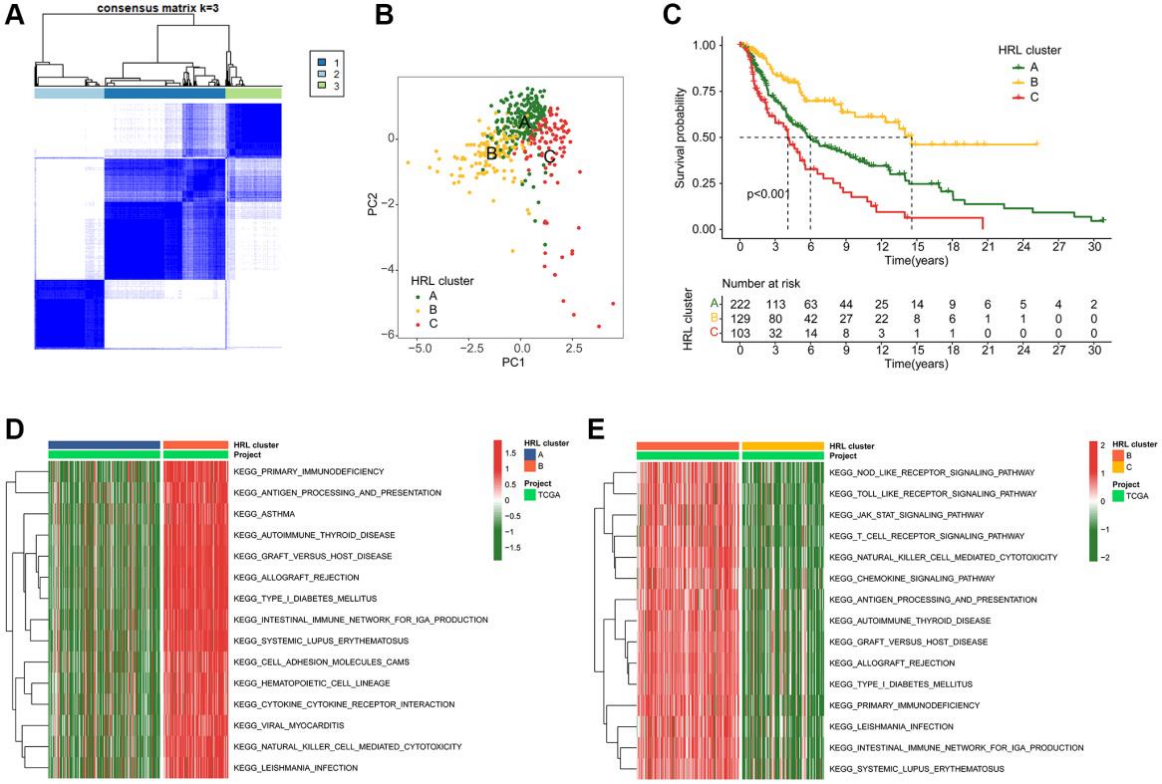
**Identification of molecular subtypes for CM.** (**A**) Unsupervised consensus clustering analysis of CM. (**B**) PCA score plot depicting cluster subgroups. (**C**) Clinical survival outcomes of CM in the clusters. (**D**, **E**) GSVA comparing KEGG signaling pathways among CM subtypes.

The molecular subtypes of CM samples were further explored using 4 prognostic HRLs. An optimal classification of K = 3 molecular subtypes was determined for CM patients using consensus clustering. This classification comprised 229, 129, 103 samples in Cluster A, Cluster B and Cluster C respectively, as depicted in the heatmap (Figure 2A). Based on the 4 prognostic HRLs, the result of principal components analysis (PCA) demonstrated a distinct separation of patients into Cluster A, Cluster B and Cluster C (Figure 2B). Kaplan-Meier survival curve analysis showed that CM patients in Cluster B had the highest overall survival (OS), which significantly different from the other two groups of patients. (Figure 2C). Differentially expressed genes (DEGs) between Cluster A and Cluster B were further enriched in immunodeficiency, antigen processing and presentation, and asthma by KEGG pathway analysis (Figure 2D). DEGs between Cluster B and C were enriched in the receptor signaling pathway, JAK-STAT signaling pathway and toll-like receptor signaling pathway (Figure 2E).

**Figure 3:**
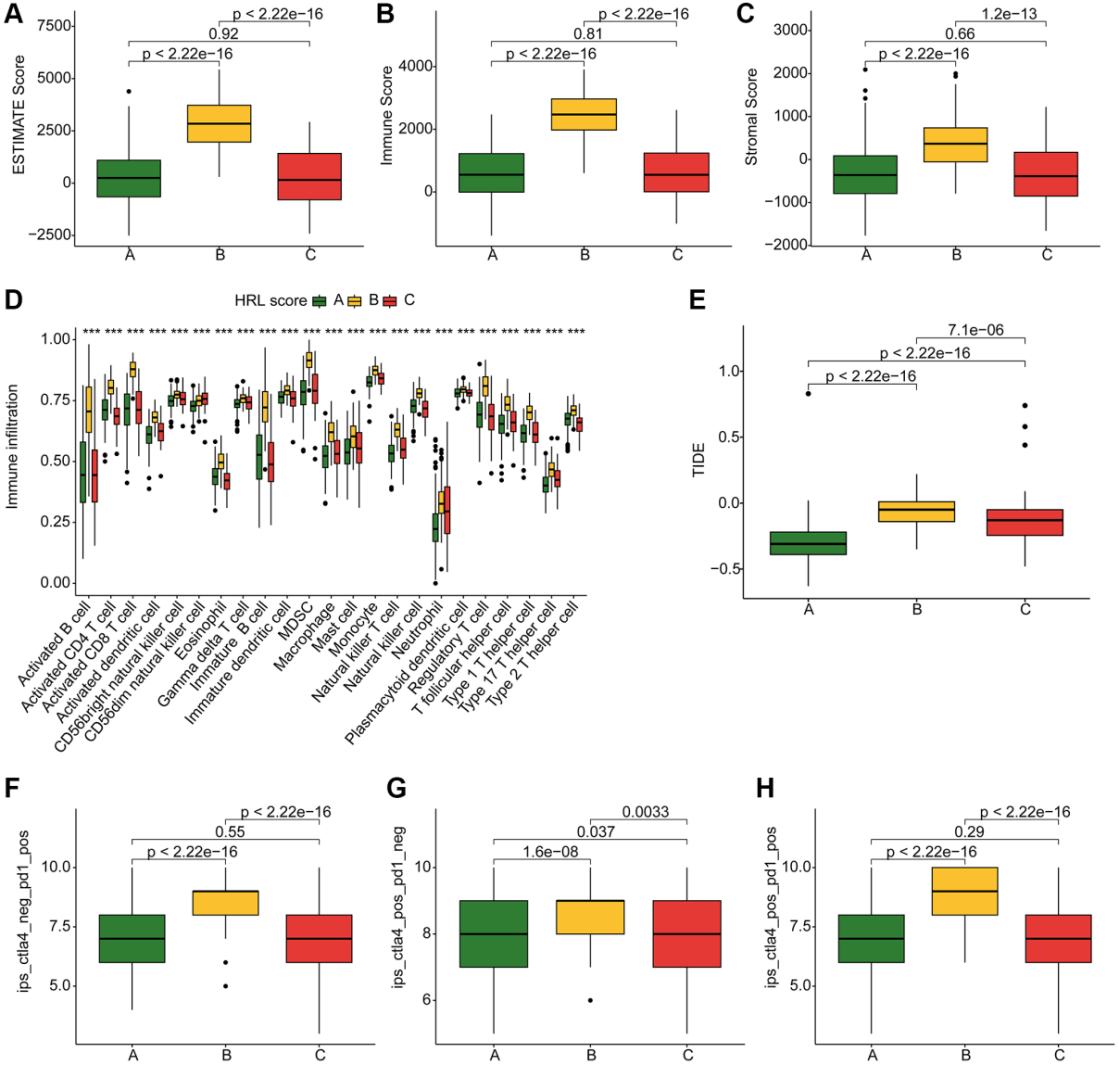
**TME landscape and immunotherapy response of CM subtypes.** (**A**–**C**) ESTIMATE score, Immune score, and Stromal score for Cluster A, B, and C. (**D**) Immune infiltration of 23 types of immune cells in Cluster A, B, and C. (**E**) TIDE score for Cluster A, B, and C. (**F**–**H**) IPS evaluation demonstrates the response of CM subtypes to PD-1 and CTLA-4.

The ESTIMATE assessment algorithm showed patients in Cluster B had the highest ESTIMATE scores, Immune scores, and Stromal scores among the three clusters (Figure 3A–3C). The results of ssGSEA algorithm showed a high level of immune cell enrichment in cluster B patients (Figure 3D). Furthermore, the results of immune function assessment indicated higher immune function scores in Cluster B patients (Figure 3E). IPS results exhibited a best response potential to anti-CTLA-4, anti-PD-1, and the combination of anti-CTLA-4/anti-PD-1 in Cluster B patients, thereby indicating a greater benefit for immunotherapy (Figure 3F–3H). In conclusion, these results provide valuable insights for future individualized precision therapy in CM patients from different subgroups.

### HRLs-based risk model development and validation

**Figure 4:**
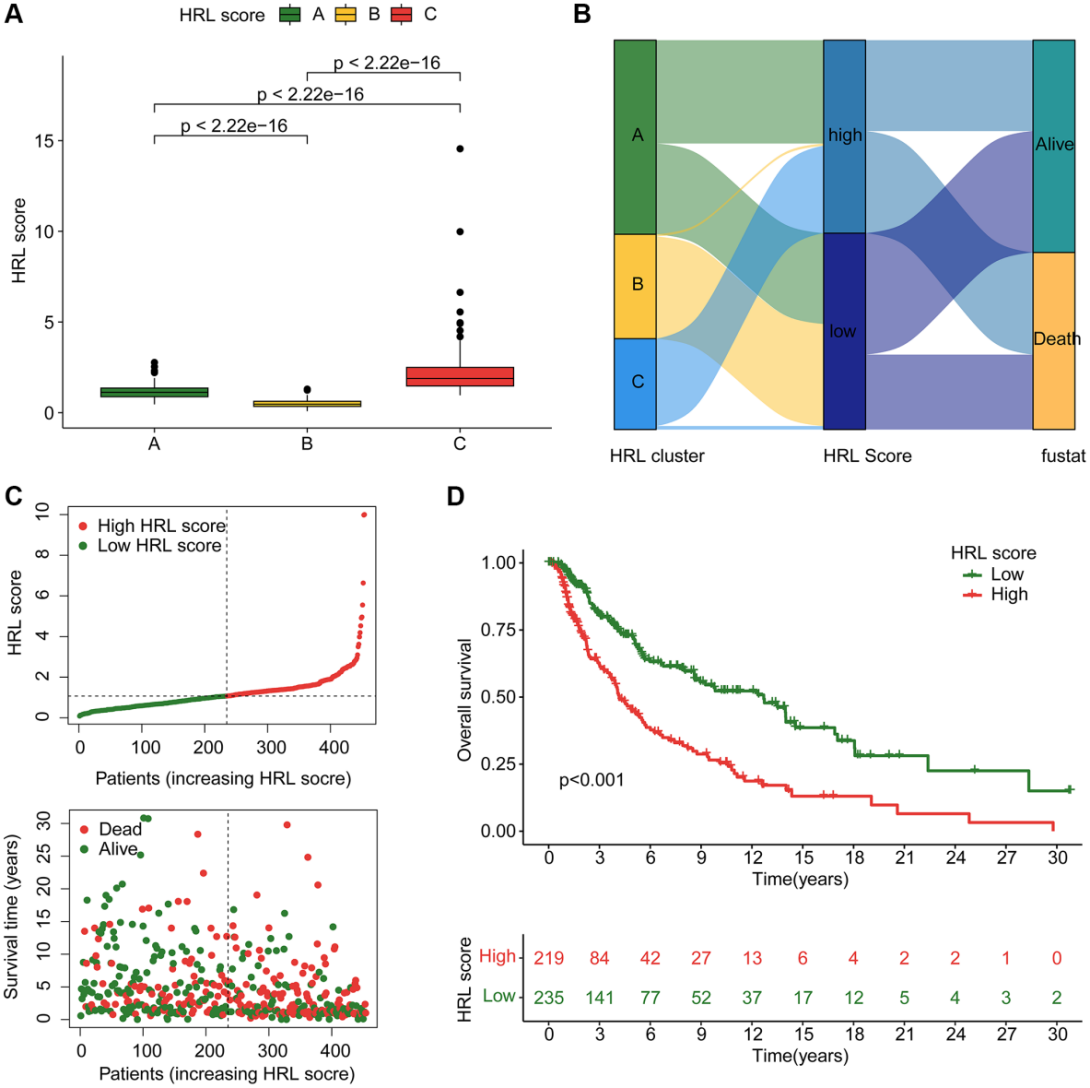
**Construction of HRLs risk model in CM.** (**A**) HRL score in CM subtypes. (**B**) Relationship between HRL score, CM cluster, HRL score, and clinical survival status. (**C**) Construction of risk model for CM. (**D**) Clinical prognostic analysis of CM samples in low-risk and high-risk groups.

A risk model was further developed based on 4 prognostic HRLs to assess clinical prognosis. Patients in Cluster C exhibited the worst clinical prognostic outcome, as indicated by the highest risk score among CM cluster subtypes (Figure 4A). The Sankey plot demonstrated the relationship among clusters, risk scores and clinical survival status (Figure 4B). The samples in the established signature were subsequently divided into training cohort (*n* = 318) and test cohort (*n* = 136) using a 7:3 classification ratio. In the entire risk cohort, the CM samples were classified into low- and high-risk groups based on the median risk score. According to the median risk score, CM samples in the entire risk cohort were divided into low-risk and high-risk groups. The results showed that the low-risk score samples had a more optimistic prognosis (Figure 4C). Kaplan-Meier analysis showed that OS rates of with low-risk score CM samples were significantly higher than that with high-risk scores (Figure 4D). Notably, the clinical survival outcomes of CM samples in the training cohort and test cohort were consistent with those of the entire cohort. CM samples categorized as low-risk had a better OS rate in comparison to the other group (Supplementary Figure 1A, 1B). Furthermore, the time-dependent ROC curves indicated that the area under the curve (AUC) for 1-, 3-, and 5-year survival was 0.726, 0.701, and 0.720 in the training group, and 0.653, 0.595, and 0.691 in the test group, respectively (Supplementary Figure 1C, 1D). In conclusion, these results validated the reliability of established HRLs based risk model in assessing clinical outcomes.

### Independent prognostic analysis of HRLs based risk model in CM

**Figure 5:**
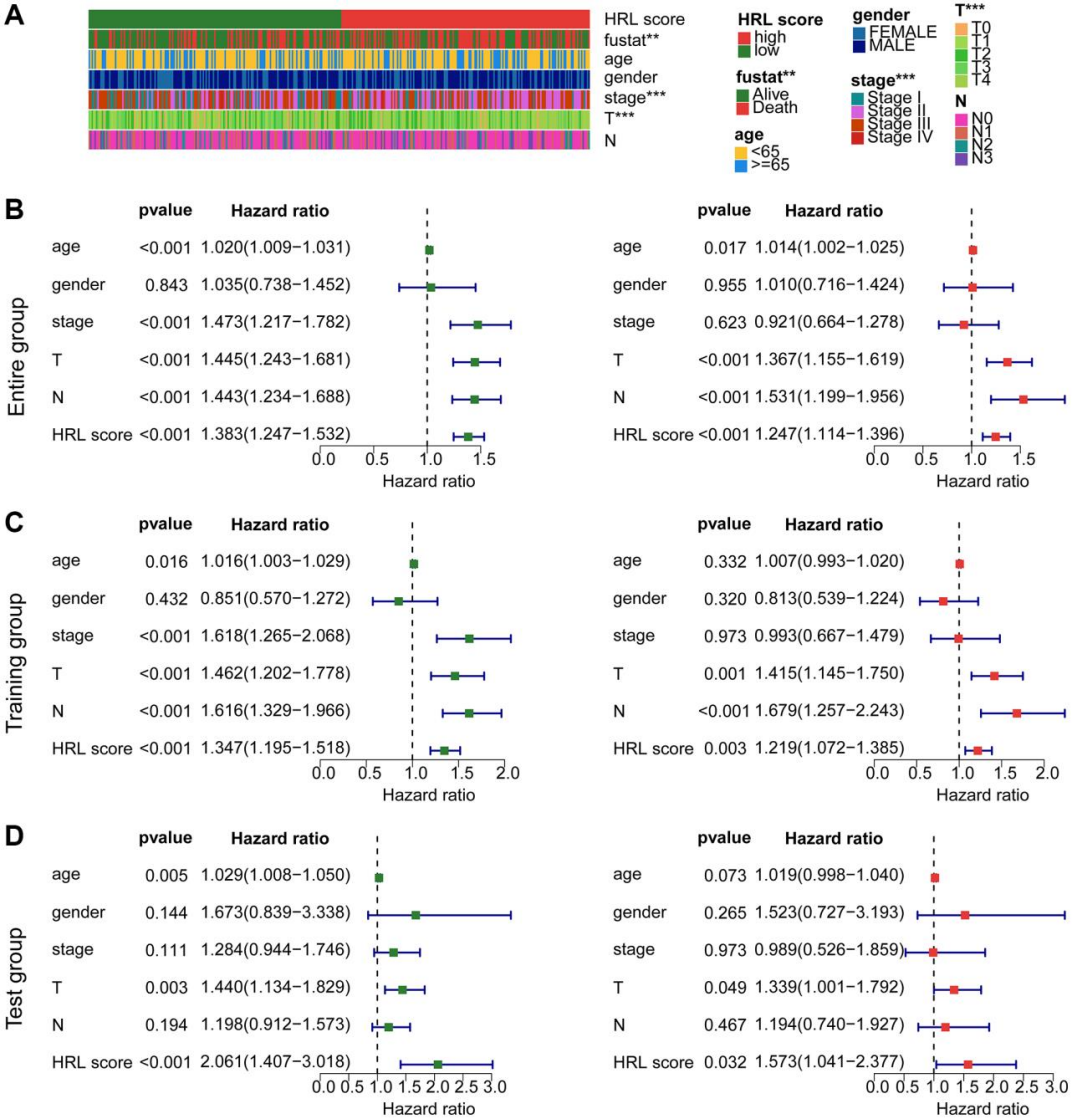
**Independent prognosis analysis of risk score and predictive ability evaluation.** (**A**) Clinicopathological characteristics of CM clusters. (**B**) Univariate and multivariate Cox analysis in the entire cohort. (**C**) Independence analysis in the training cohort. (**D**) Independence analysis in the test cohort.

The study further explored the independence of the established risk model by combining with the clinical characteristics. The results indicated a significant difference between fustat, stage (I, II, III, IV) and T stage (T0, T1, T2, T3, T4) (Figure 5A). Univariate Cox analysis in the entire cohort indicated that poor clinical prognosis was associated with the clinical characteristics including age (HR = 1.020 (1.009–1.031), *p* < 0.001), stage (HR = 1.473 (1.217–1.782), *p* < 0.001), T (HR = 1.445 (1.243–1.681), *p* < 0.001), N (HR = 1.443 (1.234–1.688), *p* < 0.001) and HRL score (HR = 1.383 (1.247–1.532), *p* < 0.001) (Figure 5B). The multivariate Cox analysis revealed that age (HR = 1.014 (1.002–1.025), *p* = 0.017), T (HR = 1.367 (1.155–1.619), *p* < 0.001), N (HR = 1.531 (1.199–1.956), *p* < 0.001) and HRL score (HR = 1.247 (1.114–1.396), *p* < 0.001) as CM independent factors (Figure 5B). The results from the training and test cohorts suggesting that the risk score could be considered as an independent prognostic indicator for CM, which exhibited better predictive value in comparison to other clinical features (Figure 5C, 5D).

### The TME landscape and immunotherapy response by risk stratification

**Figure 6:**
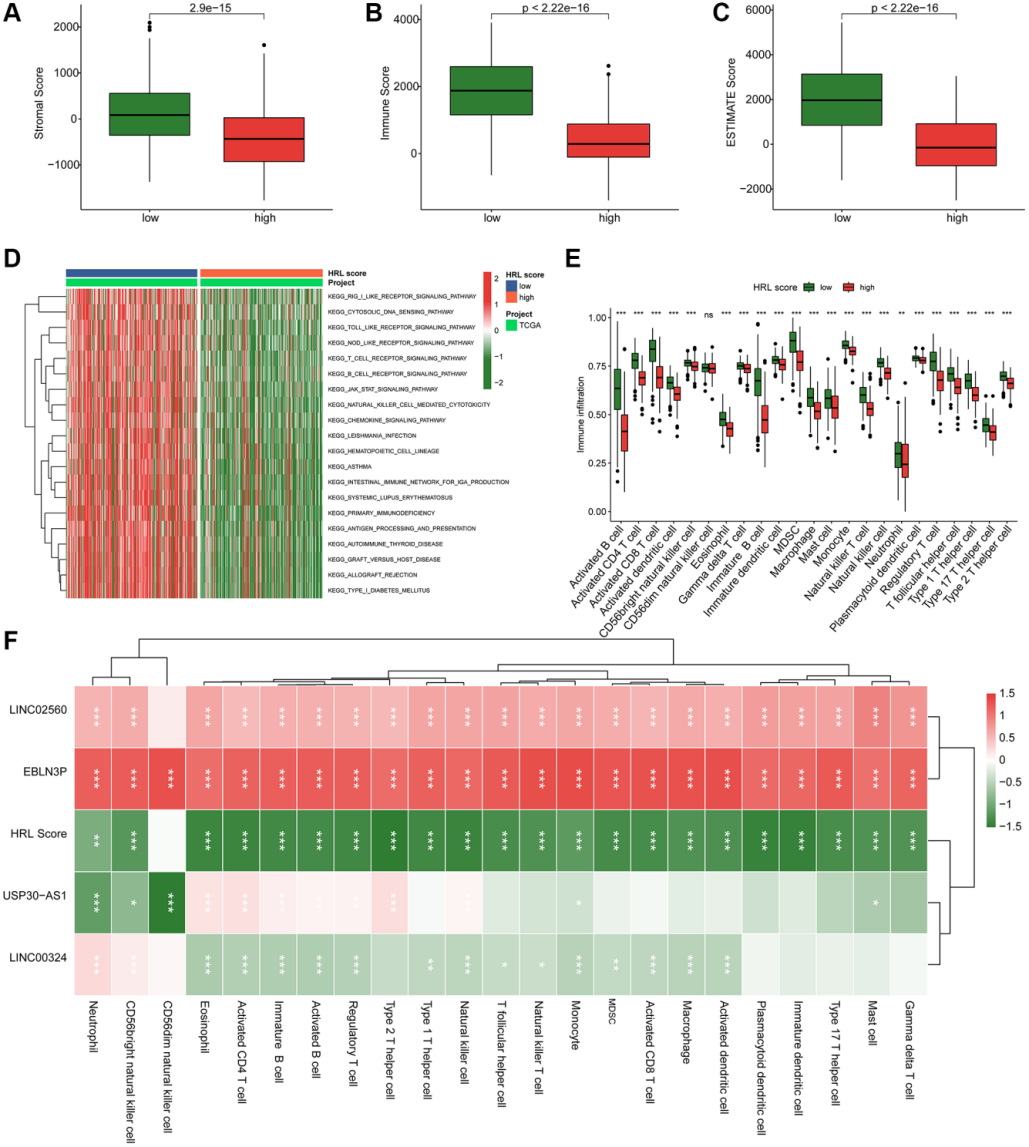
**TME landscape and immunotherapy response of CM in the risk subgroups.** (**A**–**C**) Stromal score, Immune score, and ESTIMATE score. (**D**) GSVA comparing KEGG signaling pathways between low-risk and high-risk groups. (**E**) Estimation of the proportions of 23 immune cells using ssGSEA. (**F**) Correlation analysis of LINC02560, EBLN3P, USP30-AS1, LINC00324 with HRL score and immune cells.

The tumor microenvironment (TME) landscape and immunotherapy response analysis by risk stratification were further explored. The ESTIMATE results demonstrated that high-risk score CM patients had lower stromal, immune and overall ESTIMATE scores (Figure 6A–6C). The KEGG pathway analysis indicated that DEGs by risk stratification were enriched in biological processes including cytosolic DNA sensing pathway, the rig-like receptor signaling pathway and toll-like receptor signaling pathway (Figure 6D). The immune infiltration assessment using semi-supervised gene set enrichment analysis (ssGSEA) revealed significant differences in various immune cell populations between the two groups, including activated B cells, CD4^+^ T cells, CD8^+^ T cells, and MDSCs (Figure 6E). The correlation analysis revealed a positive relationship between LINC05260 and EBLN3P expression levels and immune cells, whereas USP30-AS1, LINC00324, and the HRL score showed a negative association with immune cells (Figure 6F).

### Somatic mutation landscape and drug sensitivity analysis

**Figure 7:**
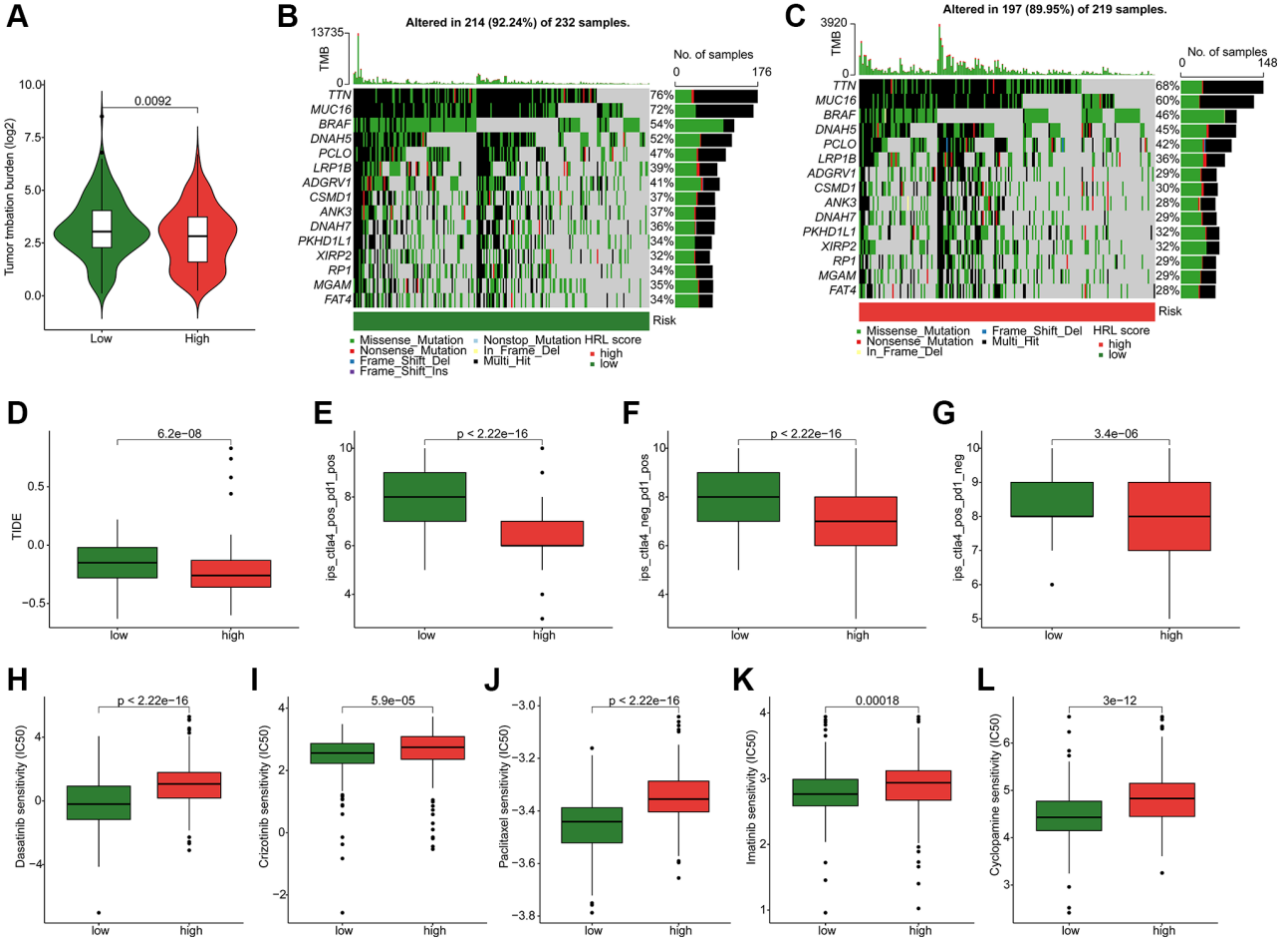
**Somatic mutation landscape and drug sensitivity in CM.** (**A**) TMB analysis. (**B**, **C**) Genetic mutation frequency in the low-risk and high-risk groups. (**D**) TIDE scores of CM patients in the low-risk and high-risk groups. (**E**–**G**) IPS results in the low-risk and high-risk groups. Distribution of IC50 values in the low-risk and high-risk groups for (**H**) dasatinib, (**I**) crizotinib, (**J**) paclitaxel, (**K**) imatinib, and (**L**) cyclopamine.

The following results showed a higher tumor mutational burden (TMB) level in low-risk group (Figure 7A). The genetic mutation landscape analysis demonstrated the somatic mutation frequency in both groups. As shown in Figure 7B, 7C, 214 (92.24%) out of 232 low-risk patients and 197 (89.95%) out of 219 high-risk patients exhibited somatic mutations. For the low-risk patients, the mutation frequencies for TTN, MUC16, BRAF, DNAH5, and PCLO were 76%, 72%, 54%, 52%, and 47%, respectively, which were higher than in the other group (Figure 7B, 7C). The TIDE results revealed that low-risk patients had higher TIDE scores (Figure 7D). Additionally, the IPS results indicated higher sensitivities to PD-1, CTLA-4, or combined treatments for low-risk patients (Figure 7E–7G). Figure 7H–7L displayed the drug sensitivity analysis results, which indicated significantly higher IC50 values of dasatinib, crizotinib, paclitaxel, imatinib, and cyclopamine in the high-risk group. These findings indicated differences in drug sensitivity by risk stratification and provided new insights into precisely targeted therapy for CM patients.

### RT-qPCR validation of selected HRLs

**Figure 8:**
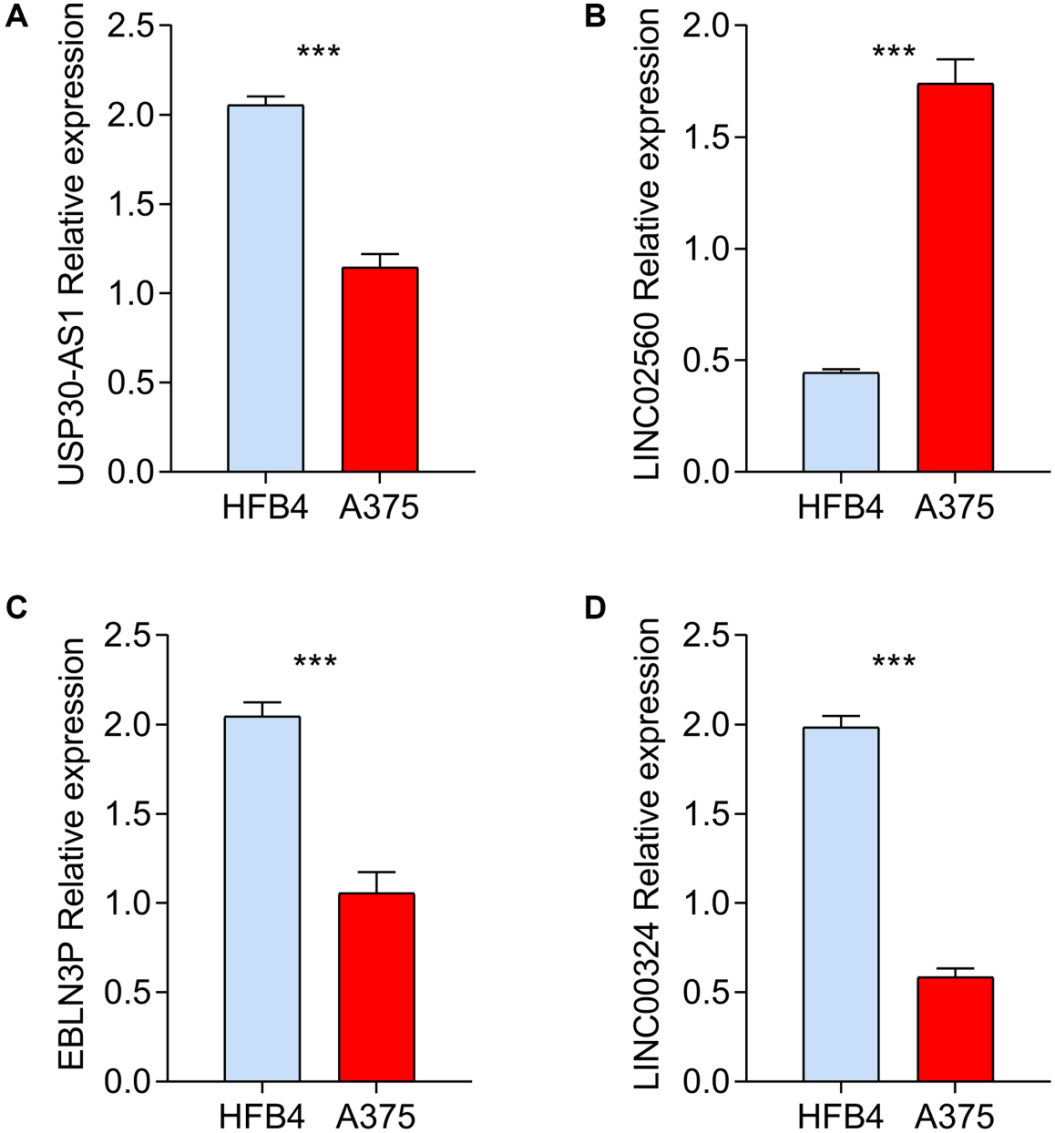
***In vitro* validation of prognostic HRLs in HFB4 and A375 cell lines.** RT-qPCR was used to test the mRNA levels of USP30-AS1 (**A**), LINC02560 (**B**), EBLN3P (**C**) and LINC00324 (**D**) in HFB4 and A375 cell lines.

We used A375 melanoma cell line for RT-qPCR to verify the bioinformatics screening results in comparison to HFB4 control cell line. As shown Figure 8A–8D, the mRNA expression levels of 4 screened HRLs according to public database showed significant differences with same tendency, which partially verified the reliability of bioinformatics results.

## DISCUSSION

In this study, we established a new HRLs-related risk model in CM and comprehensively assessed the possible causes of the difference in prognosis by risk stratification.

Although there is no direct evidence for the function of EBLN3P in CM carcinogenesis, the regulatory effect of EBLN3P on downstream targets may influence the prognosis of CM. EBLN3P has been reported to regulate the expression of U2AF homology motif kinase 1 (UHMK1) targeting miR-323a-3p [31]. As an RNA processing kinase, UHMK1 controls protein synthesis by regulating the expression and phosphorylation levels of key genes in biological processes [32]. It has been reported in melanoma patients that UHMK1 regulates metabolic reprogramming during targeted therapy through selective mRNA processing and translation, resulting in resistance to targeted therapy [33]. This may be one of the reasons for the prognostic difference by risk stratification.

Despite the lack of in-depth reports on mechanisms, USP30-AS1 has been reported to be associated with melanoma prognosis [34]. One of the main roles of USP30-AS1 is to regulate the expression level of ubiquitin-specific protease 30 (USP30) [35, 36]. USP30 is regulated by post-translational modifications and takes an active part in many cellular events such as infection, autophagy, BAX/ bak-dependent apoptosis, and tumorigenesis [37, 38]. USP30 defects lead to mitochondrial depletion, which significantly reduces the killing capacity of effector cytotoxic T lymphocytes (CTLs) [39]. Given the significance of CTLs in the development of melanoma [40], mitochondrial dysfunction caused by the USP30-AS1/USP30 axis may be an important cause.

In addition to its classic function of complex ceRNA network forming, LINC00324 can also bind RNA-binding proteins and recruit transcription factors in order to regulate multiple downstream gene expressions [41]. Although no relevant studies have been conducted in CM, LINC00324 has been shown to be overexpressed in a variety of cancer types and to be associated with pathologic features and risk stratification [42]. Unlike most tumor types, we observed a significant reduction in LINC00324 mRNA level in the A375 melanoma cell line compared to the HFB4 control cell line, which is in line with the observation in breast cancer [43]. LINC00324 affects multiple miRNA/mRNA axles and is involved in the regulation of various signaling pathways, such as miR-139-5p/IGF1R, miR-615-5p/AKT1, Mir-799-5p /STAT3, and miR-10b-5p/E-cadherin [44]. Among them, miR-10b-5p was involved in the inhibition of e-cadherin during EMT [45, 46]. By regulating miR-10b-5p, LINC00324 can promote the expression level of E-cadherin, thereby inhibiting tumor progression [46]. This may partially explain the correlation between LINC00324 and prognosis in CM.

The adaptive mechanisms by which cancer cells survive prolonged oxygen deprivation often led to the emergence of drug resistance. Its mechanisms include HIF-1α induced tight packing of genetic material, decreased mitochondrial DNA damage levels of organelles, decreased reactive oxygen species levels, and activation of pro-survival pathways [12, 47, 48]. Our results again suggest the guiding value of hypoxia related prognostic models for immune response and sensitivity to specific chemotherapy drugs. Therefore, new therapeutic strategies targeting hypoxic tumor microenvironments, including HIF-1α inhibitors, hypoxia relief, oxygen sensitive therapy, etc., have clinical application potential of CM [49]. In addition, hypoxia can inhibit CTL cytotoxic activity, thus affecting immune response [50]. Our IPS results suggest poor response to immunotherapy in the high-risk group. This was consistent with the trend of reduced levels of immune components in high-risk patients.

In summary, we establish a novel risk model related to HRLs and provide a new basis for CM risk stratification and target screening. Limited by the conditions, most of the conclusions in this paper are correlation studies rather than causation studies. Further *in vitro* and *in vivo* experiments can better reveal the effect of selected HRLs on the pathogenesis of CM in the future. Additionally, the sources of data in the database also lack of ethnic diversity and sample size. Further multi-center studies worldwide investigation will help us to know better about the role of HRLs in tumorigenesis.

## Supplementary Materials

Supplementary Figure 1

Supplementary Table 1

Supplementary Table 2
