# Supplementary material for: Identification of hypoxic-related lncRNAs prognostic model for revealing clinical prognostic and immune infiltration characteristic of cutaneous melanoma
Source: Aging (Albany NY). 2024 Feb 15;16(4):3734–49. doi: 10.18632/aging.205556 (PMC10929800; doi:10.18632/aging.205556)
Supplement: Supplementary Figure 1 [file aging-16-205556-s001.pdf]

SUPPLEMENTARY FIGURE

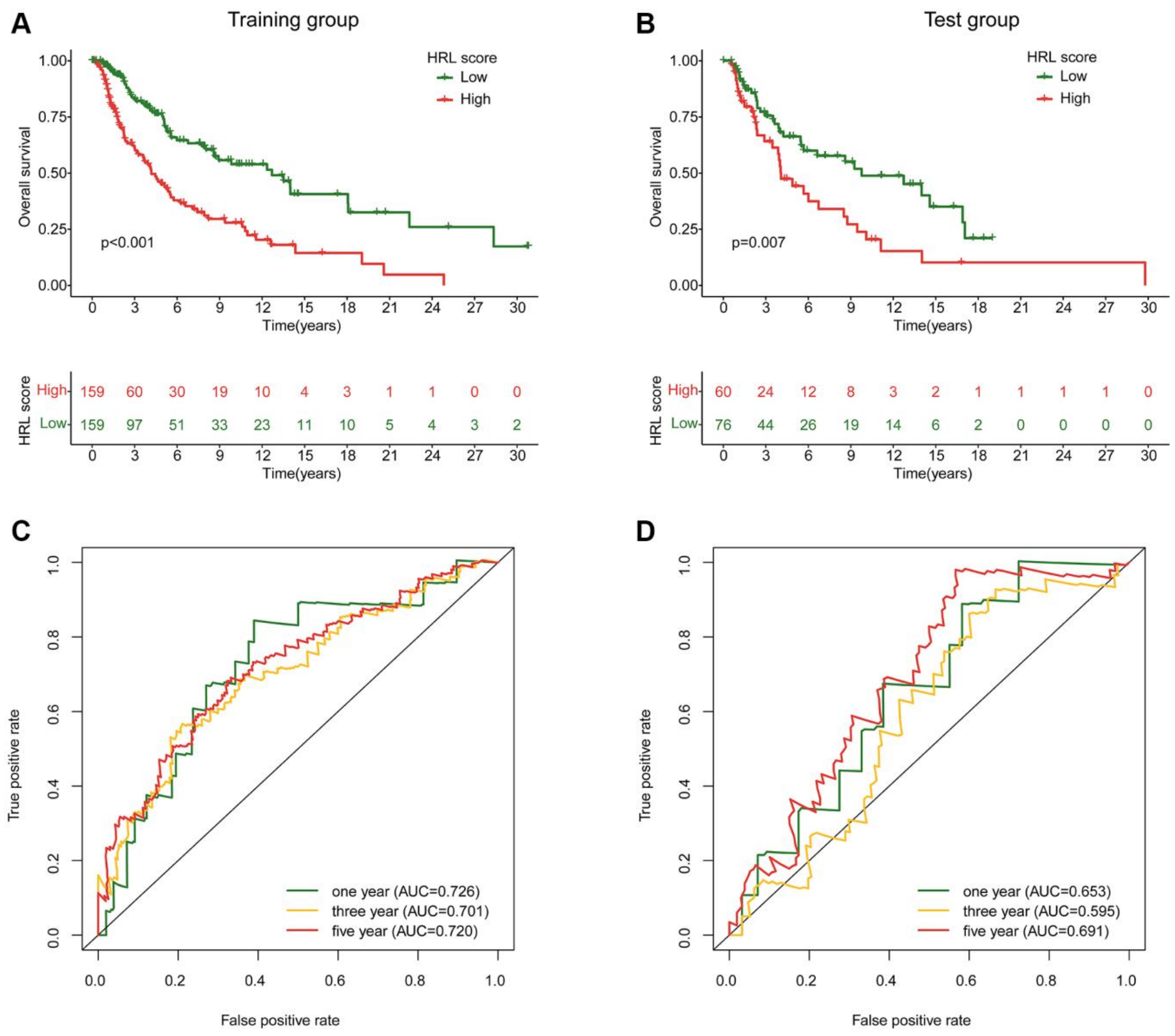

Supplementary Figure 1. Clinical prognostic analysis of CM samples in the low-risk and high-risk groups in the (**A**) Training cohort and (**B**) Test cohort. (**C**) ROC curve in the training cohort and (**D**) Test cohort.
