# Supplementary material for: Identification of hypoxic-related lncRNAs prognostic model for revealing clinical prognostic and immune infiltration characteristic of cutaneous melanoma
Source: Aging (Albany NY). 2024 Feb 15;16(4):3734–49. doi: 10.18632/aging.205556 (PMC10929800; doi:10.18632/aging.205556)
Supplement: Supplementary Table 1 [file aging-16-205556-s002.docx]

**Supplementary Table 1. The gene list of hypoxia related genes signature.**

| Hypoxia related gene symbol |
| --- |
| \| ACKR3 \| \| --- \| \| ADM \| \| ADORA2B \| \| AK4 \| \| AKAP12 \| \| ALDOA \| \| ALDOB \| \| ALDOC \| \| AMPD3 \| \| ANGPTL4 \| \| ANKZF1 \| \| ANXA2 \| \| ATF3 \| \| ATP7A \| \| B3GALT6 \| \| B4GALNT2 \| \| BCAN \| \| BCL2 \| \| BGN \| \| BHLHE40 \| \| BNIP3L \| \| BRS3 \| \| BTG1 \| \| CA12 \| \| CASP6 \| \| CAV1 \| \| CAVIN1 \| \| CAVIN3 \| \| CCN1 \| \| CCN2 \| \| CCN5 \| \| CCNG2 \| \| CDKN1A \| \| CDKN1B \| \| CDKN1C \| \| CHST2 \| \| CHST3 \| \| CITED2 \| \| COL5A1 \| \| CP \| \| CSRP2 \| \| CXCR4 \| \| DCN \| \| DDIT3 \| \| DDIT4 \| \| DPYSL4 \| \| DTNA \| \| DUSP1 \| \| EDN2 \| \| EFNA1 \| \| EFNA3 \| \| EGFR \| \| ENO1 \| \| ENO2 \| \| ENO3 \| \| ERO1A \| \| ERRFI1 \| \| ETS1 \| \| EXT1 \| \| F3 \| \| FAM162A \| \| FBP1 \| \| FOS \| \| FOSL2 \| \| FOXO3 \| \| GAA \| \| GALK1 \| \| GAPDH \| \| GAPDHS \| \| GBE1 \| \| GCK \| \| GCNT2 \| \| GLRX \| \| GPC1 \| \| GPC3 \| \| GPC4 \| \| GPI \| \| GRHPR \| \| GYS1 \| \| HAS1 \| \| HDLBP \| \| HEXA \| \| HK1 \| \| HK2 \| \| HMOX1 \| \| HOXB9 \| \| HS3ST1 \| \| HSPA5 \| \| IDS \| \| IER3 \| \| IGFBP1 \| \| IGFBP3 \| \| IL6 \| \| ILVBL \| \| INHA \| \| IRS2 \| \| ISG20 \| \| JMJD6 \| \| JUN \| \| KDELR3 \| \| KDM3A \| \| KIF5A \| \| KLF6 \| \| KLF7 \| \| KLHL24 \| \| LALBA \| \| LARGE1 \| \| LDHA \| \| LDHC \| \| LOX \| \| LXN \| \| MAFF \| \| MAP3K1 \| \| MIF \| \| MT1E \| \| MT2A \| \| MXI1 \| \| MYH9 \| \| NAGK \| \| NCAN \| \| NDRG1 \| \| NDST1 \| \| NDST2 \| \| NEDD4L \| \| NFIL3 \| \| NOCT \| \| NR3C1 \| \| P4HA1 \| \| P4HA2 \| \| PAM \| \| PCK1 \| \| PDGFB \| \| PDK1 \| \| PDK3 \| \| PFKFB3 \| \| PFKL \| \| PFKP \| \| PGAM2 \| \| PGF \| \| PGK1 \| \| PGM1 \| \| PGM2 \| \| PHKG1 \| \| PIM1 \| \| PKLR \| \| PKP1 \| \| PLAC8 \| \| PLAUR \| \| PLIN2 \| \| PNRC1 \| \| PPARGC1A \| \| PPFIA4 \| \| PPP1R15A \| \| PPP1R3C \| \| PRDX5 \| \| PRKCA \| \| PYGM \| \| RBPJ \| \| RORA \| \| RRAGD \| \| S100A4 \| \| SAP30 \| \| SCARB1 \| \| SDC2 \| \| SDC3 \| \| SDC4 \| \| SELENBP1 \| \| SERPINE1 \| \| SIAH2 \| \| SLC25A1 \| \| SLC2A1 \| \| SLC2A3 \| \| SLC2A5 \| \| SLC37A4 \| \| SLC6A6 \| \| SRPX \| \| STBD1 \| \| STC1 \| \| STC2 \| \| SULT2B1 \| \| TES \| \| TGFB3 \| \| TGFBI \| \| TGM2 \| \| TIPARP \| \| TKTL1 \| \| TMEM45A \| \| TNFAIP3 \| \| TPBG \| \| TPD52 \| \| TPI1 \| \| TPST2 \| \| UGP2 \| \| VEGFA \| \| VHL \| \| VLDLR \| \| WSB1 \| \| XPNPEP1 \| \| ZFP36 \| \| ZNF292 \| |
