# Supplementary material for: Identification of hypoxic-related lncRNAs prognostic model for revealing clinical prognostic and immune infiltration characteristic of cutaneous melanoma
Source: Aging (Albany NY). 2024 Feb 15;16(4):3734–49. doi: 10.18632/aging.205556 (PMC10929800; doi:10.18632/aging.205556)
Supplement: Supplementary Table 2 [file aging-16-205556-s003.pdf]

## SUPPLEMENTARY TABLE

**Supplementary Table 2. The primer sequences of hypoxia-related lncRNAs.**

| | | |
|---|---|---|
| LINC00324 | Forward primer | TGTGGATGACAGTGTTCGGG |
| | Reverse primer | ACGCTGACCAGAAACCGTAG |
| USP30−AS1 | Forward primer | GAACGTAGACCGCAGGACAG |
| | Reverse primer | GACGTGGTCCGTCAGCTATT |
| EBLN3P | Forward primer | TGAGGACCGAGTAGTCCTGG |
| | Reverse primer | TCCTATGCCCAGATCGTCCA |
| LINC02560 | Forward primer | CACTCTACCAGCTTGGAGCC |
| | Reverse primer | AATCAGCAGACCAGATGCCC |
